# General practitioners’ perceptions on their role in light of the NHS five year forward view: a qualitative study

**DOI:** 10.1080/17571472.2018.1455270

**Published:** 2018-04-15

**Authors:** Tara Humphrey, Karen Cleaver

**Affiliations:** a Bromley Community Education Provider Network, London, UK; b Canterbury Christ Church University, England, UK; c North Kent Community Education Provider Network, Kent, UK; d East Kent Community Education Provider Network, Kent, UK; e Greenwich University, London, UK

**Keywords:** General practitioners, primary care, leadership, community education provider networks, qualitative research

## Abstract

**Background:**

The NHS is undergoing unprecedented change, central to which is policy aimed at integrating health and social care services, resulting in the implementation of new care models. GPs are at the forefront of this change. However, there is lack of academic literature on General Practitioners’ perceptions on their role in light of the new models of care proposed by the NHS Five Year Forward View which this small-scale study begins to address.

**Objectives:**

This study aims to produce a description of how GP’s construct their current and future general practice, professional status and identify within the context of the current NHS transformation agenda.

**Methods:**

Qualitative study using semi-structured interviews and one focus group to gather the perspective of GPs (*n* = 10) working across three clinical commissioning groups in South East England.

**Results:**

While the GPs embraced the principles underpinning the new care models, they were both willing and reluctant to adopt their new roles, struggled with inter-organisational and cultural barriers and their changing professional identity.

**Conclusion:**

Multi-professional education in primary and community care could be an effective model to offer support and resources to the development of the clinical and leadership skills GPs will require to respond effectively to the transformation agenda. The emergence of community education provider networks, innovative network organisations designed to support workforce transformation through education and training, can provide the vehicle through which clinical and leadership skills training are sourced and coordinated.

## Why this matters to us?

The authors are involved with over 10 CEPNs across SE London and Kent. Since 2014. Tara Humphrey (TH) has developed and maintained close collaborative partnerships working with GP Federations and Clinical Commissioning Groups (CCGs) across London and the South East of England and has witnessed from multiple perspectives the support and resistance to new care models and the workforce transformation required to support them. Whilst the NHS is offering many leadership, coaching and financial opportunities to engage GPs in leading new models of care, GPs now require a new set of skills, understanding and support, which is supported locally. With this in mind, a qualitative study exploring General Practitioners’ perceptions of their role in light of the new models of care as proposed by the NHS Five Year Forward View was undertaken. The purpose of the study was to use the insights gained to enhance the CEPNs positioning and offering in the support and development of clinical leadership amongst GPs going forward.

## Key message

Multi-professional education in primary and community care could be an effective model to offer support, and resources to the development of the clinical and leadership skills GPs will require to respond effectively to the transformation agenda. The emergence of community education provider networks (CEPNs), innovative network organisations designed to support workforce transformation through education and training, can provide the vehicle through which clinical and leadership skills training are sourced and coordinated.

## Background

Primary care in the UK is experiencing unprecedented change with a multitude of conflicting external forces at play. The key policy driver for change arises from the Government’s proposals, set out in the Five-year and GP Forward View(5YFV/GPFV) [1–3], that the need for change in primary care is widely recognised by GPs [4,5]. In recognition that a ‘one-size fits all’ approach to care delivery is not workable*,* new models of care have been proposed [1] with two care models, specific to primary care emerging: ‘multi-speciality providers’ and ‘primary and acute care systems’. The key principles of both models are summarised in Table 1.

**Table 1.** Principles and potential functions of multi-speciality providers (MSPs) and primary and acute care systems (PACS). Adapted from NHS England (2014).

Multi-speciality providers	Primary and acute care systems
Expand skill mix and thus services offered by bringing in expertise from other services, for example, senior nurses, consultant physicians, geriatricians, paediatricians and psychiatrists to work alongside community nurses, therapists, pharmacists, psychologists, social workers. Once established, outpatient consultations and ambulatory care shifted to out of hospital settings	Potential for NHS Foundation Trusts to open their own GP surgeries with registered lists. It is envisaged this will be in areas of deprivation where GP recruitment is challenging, with safeguards to ensure that the new surgeries reinforce out-of-hospital care, rather than general practice simply becoming a feeder for hospitals still providing care in the traditional ways
Potential to run local community hospitals thereby substantially expanding diagnostic services as well as other services such as dialysis and chemotherapy	Once established, PACS could take over the running of its main district general hospital
GPs and specialists in the group credentialed to directly admit their patients into acute hospitals, with out-of-hours inpatient care being supervised by a new cadre of resident ‘hospitalists’	Ultimately, PACS could take accountability for the whole health needs of a registered list of patients, under a delegated capitated budget – similar to Accountable Care Organisations emerging in Spain, the United States, Singapore, and several other countries
Ultimately could assume delegated responsibility for managing the health service budget for their registered patients. Where funding is pooled with local authorities, a combined health and social care budget could be delegated to Multispecialty Community Providers	
May also draw on the ‘renewable energy’ of carers, volunteers and patients themselves, accessing hard-to-reach groups and taking new approaches to changing health behaviours	

From an education and workforce perspective, innovative network organisations such as Community Education Provider Networks (CEPNs), have been established to support the transformation agenda. Tasked with designing, delivering and supporting education, training and strategic workforce planning, they play a key role in a re-orientated primary and community NHS workforce [6,7].

Growing multi-professional teams, new models of care, reorganised business structures and increasing education and training opportunities, whilst having the long-term intention of increasing clinical capacity and improving access to care for patients, is colliding with workforce shortages, low workforce morale [8], disputes regarding new GP contracts [9] and increased commissioning responsibilities. Together these conflicting priorities and mounting pressures have created low morale and stress, with evidence of resistance to change and a threat to the identity of the traditional role of the GP [10].

## Methodology

An in-depth qualitative interview approach was adopted [11] to determine how GP’s are making sense of new ways of working, the objectives of the study being to produce a description of how GP’s construct their general practice, professional status and identity within the context of the Five Year Forward View and emergence of new care models. Data was collected April to September 2016.

### Selection of participants

Purposive convenience sampling was employed to recruit GPs (*n* = 10) who were practising in three neighbouring clinical commissioning groups (CCGs) in the South East of England. The GP’s were included due to their involvement with local CEPNs, facilitating ease of access to these professionals, as well as their familiarity with the key principles of the Five Year Forward View and GP Forward View.

Fifty percent of the GPs worked in urban locations, 4 in the inner-city CCG and one in a semi-rural location. The GP’s had between 2.5 and 30 years’ experience with a mean length of experience of 16 years. See Table 2 for sample characteristics.

**Table 2.** Characteristics of GP sample.

GP no	Geography	Length of experience in general practice	Gender
	Semi-rural (SR)		
	Urban (U)		
	Inner City (IC)		
1	U	25	M
2	U	30	F
3	SR	5	M
4	U	28	M
5	U	10	F
6	U	7	M
7	IC	2.5	M
8	IC	3	F
9	IC	23	F
10	IC	30	M

The GPs were approached by TH by a letter containing information about the project and were invited to respond to indicate a willingness to participate.

### Data collection

Data was collected using semi-structured interviews (*n* = 6) and one (concurrent) focus group (*n* = 4). The semi-structured interviews were conducted with GP’s working in CCGs outside of London. The focus group, conducted with four GPs working in a South London based CCG, aimed to capitalise on the interactions between a group of GPs [11] providing a balance with the semi-structured interviews. A topic guide was used to structure the interviews and focus group, the same guide used for both, to explore participants understanding of the driving forces for change within primary care, their impact on changing roles and responsibilities and examples of any measures in place to make the changing role of a GP a reality (see Table 3). Ethical approval to undertake the study was gained; informed consent was obtained prior to the interviews, interviewees were assured of anonymity. The focus group lasted 60 min; interviews lasted 30–60 min generating 295 min of data.

**Table 3.** Interview topic guide.

Topic guide
(1) In which CCG area are you based? (2) How long have you been practicing as a GP? (3) Do you hold any additional roles alongside your GP role? (4) How might the NHS 5YFV enable positive change in general practice? (5) What do you consider to be the biggest challenge in general practice in your locality? (6) Since the implementation of the 5YFV, have you seen any changes in your role as a GP? (7) What skills do you think are required to lead the new model of care? (8) How well prepared do you feel in respect of your knowledge and skills, to perform your additional role? (9) How do you think the role of the GP will evolve in the future? (10) What is the most enjoyable aspect of being a GP?

### Data analysis

• Interviews and the focus group were tape recorded with recordings transcribed verbatim.
• Data was organised and stored on NVivo (version 10).
• Data transcripts were read through to familiarise with content and were then subjected to thematic analysis using constant comparative method [12].

## Findings

Despite the challenges being experienced in general practice, the participants relished their work with patients. There was aconsensus that 5YFV provided a framework for transformation in primary care while observing that it presented challenges that needed to be addressed. There was also consensus that the role of the GP would change. Three themes emerged: attitudes towards and readiness for change, resources and the future.

### Attitudes towards and readiness for change

Although the interviewees were largely supportive of the underlying principles arising from 5YFV, they expressed concerns and varying degrees of resistance. Frustration at the volume of work, increased expectations and lack of adequate resources were expressed. Concern was also expressed that these changes could deskillGPs, and for some, the changes were deeply challenging to the culture and traditional model of general practice. For example, one GP expressed the view, borne from experience, that while the idea of collaboration was very positive and the only way forward, the attitudinal difficulties in primary care, particularly in practices which were traditionally individual units which highly value their independence, would present challenges to change*.* Similarly, another GP noted that in his locality there were many small, single-handed practices with GPs near retirement; engaging with GPs who have *one foot out of the door,* and enabling practices to collaborate when they originally chose the path of single-handed membership, and having control over how they like to work and live, is seen as a specific challenge.

While all interviewees expressed the same concerns, for some, these were alleviated through engagement with national projects which were testing what the future could look like, and as one GP observed*, I think it [*5YFV*] will enable change. Whether that is positive or negative, will depend on which GP’s you speak to (GP 6.)*

### Resources

Workload as a resource was cited as a barrier to the implementation of5YFV, the broad consensus being that the changes were creating more work and, at this moment in time, working life seemed more complex. One GP felt that the burden of administrative work for GPs was a specific issue with challenges in implementing efficient administrative systems reported.

Across all the localities GPs felt there was a lack of financial support. However, additional funds were not always seen as the answer; one GP felt that *more money to do more of the same would be wasted as there are efficiencies to be made, but more money to work differently would definitely help (GP 5)*.

GPs felt that funding was not equitably distributed, a clear frustration evident that funding of secondary and primary care needed to be more balanced; inefficiency and poor performance in secondary care was reported, with a view that even if just a small portion of savings from secondary care were redistributedinto primary care, that would make a tremendous difference.

Patient demand and expectations, as well as population change, were cited as factors influencing GP’s ability to engage with new care models due to the greater demands being placed on GPs.

### The future

All Interviewees fulfilled additional roles and held leadership positions, including:

• Networking and aligning themselves withlike-minded people (GP 5).
• Using a range of communication skills through listening, understanding, articulate the various messages to sell the vision (GP 4).
• Practicing patience (GP 9).
• Demonstrating clinical and in some cases business leadership (GP 4).
• Drawing on people’s strengths and expertise and bringing people together (GP 2).
• Increasingly thinking strategically (GP 10).
• Working collaboratively (GP 4).

However, even though engagement in these activities suggested attributes of leadership, only one GP self-identified as a leader.

There was unanimous agreement that primary care would change, with a greater emphasis on the development of multi-professional teams which might include GPs with urgent and emergency care skills, practice nurses/nurse practitioners, physicians’ associates, social workers, mental health practitioners, practice/business managers, patient educators and care navigators. There was aconsensus that the introduction of new roles would reduce continuity of care, a component of practice highly valued by the interviewees. Interviewees agreed that GPs of the future would increasingly require communication and negotiation skills, entrepreneurial flair, with skills in bid writing and tendering.

## Limitations of the study

No qualitative studies have been located which examine how GP’s are responding to and engaging with new care models and associated agenda arising from 5YFV. The small sample size means that comparisons cannot be made, but, the descriptions arising from the interviews and focus group provide an indication of how this group of GPs are constructing their changing practice, status and identity, descriptions which may transfer to other GP’s current experiences.

## Comparison with existing literature

The interviewees shared a vision of how 5YFV could create opportunities. However, scepticism, resistance, and frustration with the current systems and structures, whilst trying to manage their growing workload, was apparent. Unsurprisingly, the perceptions of the GP’s mirror findings from GP surveys conducted by the BMA [8,13], with continuity of care, trust and confidentially essential factors for general practice; increased core funding, longer consultations and reduction in bureaucracy necessary factors that would assist in delivering care. Workload was seen as a significant barrier to implementing new care models.

Changes across the NHS are being driven by policy in response to mainly economic imperatives, leading to system re-design and change, adopting a largely top-down approach. Changing a deeply fragmented system which aims to bring together independent general practices to work more collaboratively across agenciesis potentially challenging; trying to align different professional cultures, work patterns, quality indicators, funding streams, amongst others, is arguably increasing the complexity tenfold. Thus, new models of care require a more sophisticated understanding of working with patients in a different way and on different levels [14].

The extent to which GPs are engaged with change arising from 5YFV is empiricallyunknown. Results from this small-scale qualitative study suggest that the participating GPs were at different stages within the ‘transition curve of change’[15]. The NHS Change Model [16] acknowledges that adequate system drivers, incentives, and processes need to be in place to successfully implement change. Findings from this study indicate that while drivers and incentives, in terms of engagement with new care models, and the potential funding aligned with these may exist, the day-to-day workload of GP’s coupled with difficulties in GP recruitment in the study sites, could mitigate against successful adoption of new care models and the system change required. Moreover, and perhaps more challenging, a change of professional culture is required, with GP’s recognising that to move forward, their roles will need to change. Thus their reservations about changes to their role and associated identity threat [10] need to be addressed.

To achieve the vision articulated through 5YFV requires individuals able to work across professional and service/agency boundaries. GPs will need to fulfil the role of system leaders, taking collective responsibility, building alliances and collaborations by engaging their peers and many others in working towards a better future, and in so doing they need to be resilient to the chaos that may initially ensue [17].

## Conclusion

The implementation of new care models and associated new ways of working is generating the need for cultural change within primary care. Although national general practice improvement programmes exist, applicability at a local level is important. Embedded within primary care and working closely with GP Federations and CCGs, CEPNs understand first-hand the local challenges and opportunities. CEPNs can serve as an effective model to offer local and bespoke support in partnership with CCG’s, GP Federations and newly establishing providers. CEPNs are uniquely positioned to help shape education for GPs and the wider general practice workforce that is locally applied and contextualised. Training programmes to develop local clinical leadership capabilities could include:

• Financial management
• Leadership support
• Increased communication across professional and organisational boundaries
• Workforce and succession planning
• Change management and organisational development
• Skills gap analysis
• Migrant health training and support

## Declarations

Ethical approval to undertake the study was obtained through Canterbury Christ Church University Research Ethics Committee.

## Disclosure statement

No potential conflict of interest was reported by the authors.

## References

[1] NHS England Care Quality Commission, Health Education England, Monitor, Public Health England, Trust Development Authority. Five Year Forward View; 2014 [cited 2015 Sep 6]. Available from: https://www.england.nhs.uk/wp-content/uploads/2014/10/5yfv-web.pdf

[2] NHS England Delivering the forward view: NHS planning guidance 2016. 17–2020; 2015 [cited 2016 Dec 1]. Available from: https://www.england.nhs.uk/wp-content/uploads/2015/12/planning-guide-16-17-20-21.pdf

[3] NHS England General practice forward view; 2016 [cited 2016 Aug 6]. Available from: https://www.england.nhs.uk/gp/gpfv/

[4] Thomas P Comprehensive primary health care: a new phase?. London J Primary Care. 2008;1:87–89. DOI:10.1080/17571472.2008.11493216 PMC4212746 25949566

[5] The Royal College of General practitioners A vision for general practice in the future NHS; 2012 [cited 2015 Sep 6]. Available from: http://www.rcgp.org.uk/campaign-home/~/media/Files/Policy/A-Z-policy/The-2022-GP-A-Vision-for-General-Practice-in-the-Future-NHS.ashx

[6] Health Education South London Health Education South London Delivery Plan 2014/15; 2014 [cited 2016 Aug 6]. Available from: http://southlondon.hee.nhs.uk/2014/06/17/delivery-plan-20142015-published/

[7] Ahluwalia S, Tavabie A, Spicer J, Chana N Community-based education providers network: an opportunity to unleash the potential for innovation in primary care education. Edu Primary Care. 2013;24:394 10.1080/14739879.2013.11494207 24196593

[8] British Medical Association National Survey of GPs, Full Report; 2015 [cited 2016 Aug 27]. Available from: file:///C:/Users/tarah/Downloads/Future%20of%20general%20practice%20full%20survey%202015%20(1).pdf

[9] The Medical Portal Junior doctor contract: what you need to know; 2016 [cited 2016 Aug 27]. Available from: https://www.themedicportal.com/junior-doctor-contract-what-you-need-to-know/

[10] Setchell J, Leach LE, Watson BM, Hewett DG Impact of identity on support for new roles in health care: a language inquiry of doctors’ commentary. J Lang Soc Psychol. 2015;34(6):672–686. 10.1177/0261927X15586793

[11] Pope C, Mays N Qualitative research in health care. 3rd ed [e-book] Malden, MA: Blackwell; 2006.

[12] Braun V, Clarke V Using thematic analysis in psychology. Qual Res Psychol. 2006;3:77–101. 10.1191/1478088706qp063oa

[13] British Medical Assoication Survey of GPs in England; 2016 [cited 2017 Feb 9]. Available from: https://www.bma.org.uk/collective-voice/influence/key-negotiations/training-and-workforce/urgent-prescription-for-general-practice/key-issues-survey

[14] Burch T Mind the gap! Conflict and cohesion in primary care delivery models. London J Primary Care. 2015;6:43–45. DOI:10.1080/17571472.2014.11493414 PMC4235357 25949714

[15] Young A, Lockhart T A cycle of change: the transition curve; 1995 [cited 2016 Aug 28]. Available from: http://www.ucd.ie/t4cms/Transition%20Curve%20Cranfield%20Article.pdf

[16] NHS Improvement An introduction to the NHS change model. London [cited 2016 Aug 28]. Available from: http://webarchive.nationalarchives.gov.uk/20160506160341/http://www.nhsiq.nhs.uk/capacity-capability/change-model.aspx

[17] Timmins N The practice of system leadership: being comfortable with chaos; 2015 [cited 2016 Aug 28]. Available from: http://www.kingsfund.org.uk/sites/files/kf/field/field_publication_file/System-leadership-Kings-Fund-May-2015.pdf

